# Supplementary material for: Maximum entropy methods for extracting the learned features of deep neural networks
Source: PLoS Comput Biol. 2017 Oct 30;13(10):e1005836. doi: 10.1371/journal.pcbi.1005836 (PMC5679649; doi:10.1371/journal.pcbi.1005836)
Supplement: S1 Text — (DOCX) [file pcbi.1005836.s001.docx]

Text S1. Details of Neural Network Layers

# Description of layer types

We here give a brief description of the functions calculated by the artificial neural network (ANN) layers employed in Applications 1-3.

# Input layer:

The input layer represents the network input as a real-valued array. Applications of ANNs in genomics frequently employ a one-hot representation in which a length L genomic sequence is represented as an $L\times4$ array. Each row corresponds to a base position and has a single 1 entry indicting the nucleotide content of the base, with other row entries equal to zero. Other representations of sequence inputs are possible. For example, the ANN in Application 3 uses an input layer representation constructed by first representing sequences as one-hot arrays and then subtracting from each column the genome wide frequency, *f*, of the corresponding nucleotide and finally dividing this difference by $\sqrt{f(1-f)}$ (Methods: Application 3).

# Fully connected layers:

The vector $\boldsymbol{a}^{\boldsymbol{i}}$ of unit activations of a fully connected layer indexed by *i*  is the result of composing an affine transformation of the preceding layer activations with a non-linear operation:

(S1.1.1)

$\boldsymbol{a}^{\boldsymbol{i}}\boldsymbol{=\sigma}( W\boldsymbol{a}^{\boldsymbol{i-1}}\boldsymbol{+b )}$,

where$W\boldsymbol{a}^{\boldsymbol{i-1}}$ denotes multiplication of the matrix *W* of weights for layer *i* with the vector of activations $\boldsymbol{a}^{\boldsymbol{i-1}}$ of the preceding layer, $\boldsymbol{b}$ is a bias term for layer *i*, and $\boldsymbol{\sigma}$**( )** denotes element-wise application of a non-linear function (also called an activation function). Applications 1-3 used two non-linear functions for $\boldsymbol{\sigma}$**:** Rectified-linear (ReLU) units use $\boldsymbol{\sigma}\left( \boldsymbol{z} \right)\boldsymbol{=}max(0, z)$. Sigmoid units use $\boldsymbol{\sigma}\left( \boldsymbol{z} \right)\boldsymbol{=}\frac{\boldsymbol{1}}{\boldsymbol{1+}\boldsymbol{e}^{\boldsymbol{-z}}}$.

# Convolutional layers:

Convolutional layers are defined by a collection of *N* convolutional filters ${\{\left( W^{i} , b^{i} \right)\}}_{i=1}^{N}$ where $W^{i}$ and $b^{i}$are the weight matrices and biases for the filter. Each filter is composed of units with the same $W^{i}$ and $b^{i}$, but receive different local patches of preceding layer activations as input. In our models, the preceding layer is the $L\times4$ input array, so that $W^{i}$ is an $h\times4$ matrix where *h* is the number of base pairs (bps) considered by the unit. The activation $a$ of a unit sensitive to a sequence interval starting at position *p* is

(S1.1.2)

$$a=\sigma\left( \sum_{j=0}^{h-1} \sum_{k=1}^{4} W_{j,k}^{i}x_{p+j, k}+b^{i} \right)$$

where $x_{p+j, k}$ denotes entry of the $\left( p+j \right)^{th}$ row and $k^{th}$ column of the array representation of the input sequence, and where $\sigma$ denotes either a ReLU or sigmoid function. A vector of filter activations (also called a feature map) has elements given by (S1.1.2) for values of *p* starting with $p =0$ and incrementing by a common integer called the stride of the layer. The *N* filter activation vectors are concatenated before being passed to the subsequent fully connected layer of our models.

# Output Layers:

Application 1 employs a single output unit with full connection to the penultimate layer and a signmoid non-linear function. Outputs greater than 0.5 indicate classifcation to class 1; outputs less than 0.5 indicate classification to class 0.

Applications 2 and 3 use a two unit output layer with a softmax activation function. The softmax function, for classification to one of K classes, yields a length K vector with component *j* given by

(S1.1.3)

$$\sigma\left( \boldsymbol{z} \right)_{j}= \frac{e^{z_{j}}}{\sum_{k=1}^{K} e^{z_{k}}}$$

where $z_{k}$ denotes the the *k^th^* entry resulting from affine transformation of the vector $\boldsymbol{\Phi}$ of penultimate layer activations. That is,

(S1.1.4)

$$z_{k}=\sum_{i=1}^{M} w_{k,i}\Phi_{i}+b_{k}$$

where (following the notation of “Results: Constructing an input-specific contrained maximum entropy distribution”) $w_{k,i}$ is the weight of connection of the *i^th^* penultimate unit to output unit *k*, $\Phi_{i}$ is the activation of the *i^th^* unit in the penultimate layer with *M* units, and $b_{k}$ is the bias of ouput unit *k*.

# Motivation for the distance metric weights in multiclass classification

ANNs performing multiclass classification among *K* classes typically encode the predicted class probabilities by using *K* output units, one for each class, with their activations calculated from softmax functions acting on affine-transformed penultimate units (S1.1.3). The softmax function ensures that class probabilities are normalized to 1. For network input $\boldsymbol{x}_{\boldsymbol{0}}$, the log ratio of predicted class 0 to class *k* ($k\in\{1,\ldots K-1\}$ ) probabilities is

$$\log\left( \frac{\mathbb{P}\left( class 0 \right|\boldsymbol{x}_{\boldsymbol{0}} )}{\mathbb{P}\left( class k \right|\boldsymbol{x}_{\boldsymbol{0}} )} \right)=\log\left( \frac{e^{z_{0}}}{e^{z^{k}}} \right)$$

where $z_{k}$ is as defined in (S1.1.3). Substituting equation (S1.1.4) yields

(S1.2.1)

$$\log\left( \frac{\mathbb{P}\left( class 0 \right|\boldsymbol{x}_{\boldsymbol{0}} )}{\mathbb{P}\left( class k \right|\boldsymbol{x}_{\boldsymbol{0}} )} \right)= \left( \sum_{i=1}^{M} \left( w_{0,i}-w_{k,i} \right)\Phi_{i}\left( \boldsymbol{x}_{\boldsymbol{0}} \right) \right)+b_{0}-b_{k}.$$

Thus, the magnitude of the difference $w_{0,i}-w_{k,i}$ is a natural choice for measuring the extent to which features encoded in the penultimate activations $\Phi_{i}$ allow the network to identify $\boldsymbol{x}_{\boldsymbol{0}}$ as belonging to class 0 rather than class *k.*

(S1.2.2)
