# Supplementary material for: Maximum entropy methods for extracting the learned features of deep neural networks
Source: PLoS Comput Biol. 2017 Oct 30;13(10):e1005836. doi: 10.1371/journal.pcbi.1005836 (PMC5679649; doi:10.1371/journal.pcbi.1005836)
Supplement: S2 Text — (DOCX) [file pcbi.1005836.s002.docx]

S2 Text. Mathematical Derivations.

# Derivation of equation (2)

We solve the constrained minimization problem (1) by seeking critical points of the Lagrange function $\mathcal{L}$ given by

(S2.1.1)

$$\mathcal{L=}\sum_{\boldsymbol{x}\in X} p_{\boldsymbol{x}_{\boldsymbol{0}}}\left( \boldsymbol{x} \right)\log\left( \frac{p_{\boldsymbol{x}_{\boldsymbol{0}}}\left( \boldsymbol{x} \right)}{q\left( \boldsymbol{x} \right)} \right)+ \beta\left( \sum_{\boldsymbol{x}\in X} p_{\boldsymbol{x}_{\boldsymbol{0}}}\left( \boldsymbol{x} \right)d\left( \boldsymbol{\Phi}\left( \boldsymbol{x}_{\boldsymbol{0}} \right)\boldsymbol{, \Phi}\left( \boldsymbol{x} \right) \right) -D \right)-\alpha\left( \sum_{x\in X} p_{\boldsymbol{x}_{0}}\left( x \right) -1 \right)$$

where *X* denotes the set of all length L sequences, $p_{\boldsymbol{x}_{\boldsymbol{0}}}(\boldsymbol{x)}$ and $q\left( \boldsymbol{x} \right)$ denote the probabilities of sequence $\boldsymbol{x}$ under PMFs $p_{\boldsymbol{x}_{\boldsymbol{0}}}$ and $q$, and $\beta$ and $\alpha$ are Lagrange multipliers associated with the constraints. Setting the partial derivatives $\frac{\partial\mathcal{L}}{\partial p_{\boldsymbol{x}_{\boldsymbol{0}}}(\boldsymbol{x)}}$ to zero, and solving for $p_{\boldsymbol{x}_{\boldsymbol{0}}}(\boldsymbol{x)}$ gives:

(S2.1.2)

$$p_{\boldsymbol{x}_{\boldsymbol{0}}}(\boldsymbol{x)=}\frac{e^{-\beta d(\boldsymbol{\Phi}\left( \boldsymbol{x}_{\boldsymbol{0}} \right)\boldsymbol{, \Phi}\left( \boldsymbol{x} \right)\boldsymbol{)}} q\left( \boldsymbol{x} \right)}{Z}$$

where $Z\equiv e^{1-\alpha}$ is the normalization constant for the probability distribution.

As stated in the main text, we choose $q\left( \boldsymbol{x} \right)$ to be a product of identically distributed single nucleotide distributions:

$$q\boldsymbol{(x)=}\prod_{\boldsymbol{i}=1}^{L} \frac{e^{\mu I(x_{i})}}{2(1+e^{u})}$$

where $I(x_{i})$ is the indicator function for a G or C nucleotide at position $i$, and $\mu$ controls the average GC content of a sequence position. To fix $\mu,$ we let $c$ denote the genome GC content and require

(S2.1.4)

(S2.1.3)

$$c=E_{q}[ I\left( x_{i} \right)]$$

where $E_{q}$ denotes expectation with respect to $q$ and $i$ is an arbitrary sequence positon. Solving for $\mu$ gives

(S2.1.5)

$$\mu= \log\left( \frac{c}{1-c} \right)$$

Substituting $q\boldsymbol{(x)}$ given by (S2.1.3) with this value of $\mu$ into (S2.1.2) and absorbing the denominator of $q\left( \boldsymbol{x} \right)$ into $Z$, we obtain equation (2).

# Approximating the decay of $\boldsymbol{f(v)}$

To estimate the decay of $f(v)$ from its maximum in terms of quantities measurable from our MaxEnt samples of $p_{\boldsymbol{x}_{\boldsymbol{0}}}$, we take logs of equation (5) to obtain

(S2.2.2)

(S2.2.1)

$$\log f\left( v \right)=\log\left( \sum_{\mathbf{x}\in X_{v}} q\left( \boldsymbol{x} \right)e^{-\beta d\left( \boldsymbol{\Phi}\left( \boldsymbol{x}_{\boldsymbol{0}} \right)\boldsymbol{, \Phi}\left( \boldsymbol{x} \right) \right)} \right)- \log\left( \sum_{\mathbf{x}\in X_{v}} q\left( \boldsymbol{x} \right) \right)$$

$$\log f\left( v \right)=\log\left( \sum_{\mathbf{x}\in X_{v}} \frac{q(\boldsymbol{x})e^{-\beta d\left( \boldsymbol{\Phi}\left( \boldsymbol{x}_{\boldsymbol{0}} \right)\boldsymbol{, \Phi}\left( \boldsymbol{x} \right) \right)}}{Z} \right)- \log\left( \sum_{\mathbf{x}\in X_{v}} q\left( \boldsymbol{x} \right) \right)+log(Z)$$

The first term of (S2.2.2) is the log of the marginal distribution of the input-wide feature $V(\boldsymbol{x})$ when$\boldsymbol{x}$ is distributed according to $p_{\boldsymbol{x}_{\boldsymbol{0}}}$. We denote this marginal distribution as

(S2.2.3)

$$\mathbb{P}_{p_{\boldsymbol{x}_{\boldsymbol{0}}}}\left( V=v \right)\equiv\sum_{\mathbf{x}\in X_{v}} \frac{q\left( \boldsymbol{x} \right)e^{-\beta d\left( \boldsymbol{\Phi}\left( \boldsymbol{x}_{\boldsymbol{0}} \right)\boldsymbol{, \Phi}\left( \boldsymbol{x} \right) \right)}}{Z} .$$

Similarly, the second term is the log of the marginal distribution of the input-wide feature $V(\boldsymbol{x})$ when $\boldsymbol{x}$ is distributed according to $q$. We denote this marginal distribution as

(S2.2.4)

$$\mathbb{P}_{q}\left( V=v \right)\equiv\sum_{\mathbf{x}\in X_{v}} q\left( \boldsymbol{x} \right).$$

Our choice of *q* as a product of identical single nucleotide distributions (equation (S2.1.3)) implies that when $\boldsymbol{x}$ is distributed according to *q*, $V(\boldsymbol{x})$ is a linear combination of the independent random variables $I_{i}(x_{i})$ with weights $c_{i}$(equation (4) main text). Assuming this linear combination involves a sufficient number of weights of roughly the same magnitude, we can apply the Lindeberg version of the Central Limit Theorem to approximate

$$\mathbb{P}_{q}\left( V=v \right)\approx\frac{1}{\sqrt{2\pi\sigma^{2}}}e^{-\frac{1}{2\sigma^{2}}\left( v- \left\langle V \right\rangle_{q} \right)^{2}}$$

where $\left\langle V \right\rangle_{q}$ and $\sigma^{2}$ denote the mean and variance of $V(\boldsymbol{x})$, respective, when $\boldsymbol{x}$ is distributed according to *q*. These moments can be calculated directly from the simple form for *q* (equation (S2.1.3)).

(S2.2.5)

Approximation (S2.2.5) treats $\mathbb{P}_{q}\left( V=v \right)$ as a smooth function of $v$. To approximate the decay of $f\left( v \right)$, we also treat $\mathbb{P}_{p_{\boldsymbol{x}_{\boldsymbol{0}}}}\left( V=v \right)$ as a smooth function of $v$ and expand $\log f\left( v \right)$ in a second order Taylor Series about the value $v^{*}$ that maximizes $\log f\left( v \right):$

(S2.2.6)

$\log\left( f\left( v \right) \right)\approx\log\left( f\left( v^{*} \right) \right)+\frac{1}{2}\left( \frac{1}{\sigma^{2}}+ \left. \frac{d^{2}}{dv^{2}}\log\left( \mathbb{P}_{p}\left( V=v \right) \right) \right|_{v=v^{*}} \right)(v-{v^{*})}^{2}$.

The 1^st^ order term is zero since we are expanding about a maximum, and we have substituted $\frac{-1}{\sigma^{2}}$ for $\frac{d^{2}}{dv^{2}}\log\left( \mathbb{P}_{p}\left( V=v \right) \right)$. Truncation of higher order terms is justified if the distribution $\mathbb{P}_{p_{\boldsymbol{x}_{\boldsymbol{0}}}}\left( V=v \right)$ is approximately normal, a condition which can be checked directly by estimating the distribution of $V(\boldsymbol{x})$ from MCMC samples. Moreover, in this case we can estimate

(S2.2.7)

$$\frac{-1}{s^{2}}=\left. \frac{d^{2}}{dv^{2}}\log\left( \mathbb{P}_{p_{\boldsymbol{x}_{\boldsymbol{0}}}}\left( V=v \right) \right) \right|_{v=v^{*}}$$

where $s^{2}$ is the variance of $V(\boldsymbol{x})$ under the distribution $p_{\boldsymbol{x}_{\boldsymbol{0}}}$, estimated from MCMC samples. With this estimate and under the above approximations, we obtain:

$$f\left( v \right)\propto e^{-\frac{1}{2}\left( \frac{1}{s^{2}} - \frac{1}{\sigma^{2}} \right)\left( v-v^{*} \right)^{2}}$$

which is equation (6).
