# Supplementary material for: Maximum entropy methods for extracting the learned features of deep neural networks
Source: PLoS Comput Biol. 2017 Oct 30;13(10):e1005836. doi: 10.1371/journal.pcbi.1005836 (PMC5679649; doi:10.1371/journal.pcbi.1005836)
Supplement: S3 Text — (DOCX) [file pcbi.1005836.s003.docx]

**Text S3. Details for Implementing DeepLIFT and Saliency Map**

# Accession and use of DeepLIFT code

We used DeepLIFT version 0.4.0 from <https://github.com/kundajelab/deeplift>. The main output of DeepLIFT interpretation described in [1] are arrays of contributions of input neurons (encoding genomic sequence) to output neurons encoding the classification of a trained artificial neural network (ANN). We converted Keras sequential models [2] (used for training) to DeepLIFT models by passing the “nonlinear_mxts_mode = NonlinearMxtsMode.DeepLIFT” argument to the DeepLIFT model conversion function. Since DeepLIFT contribution arrays have the same shape as the network input, i.e. (sequence Length) x 4 for the standard one-hot encoding scheme, DeepLIFT assigns scores to each sequence position by summing over contributions of input neurons associated with a fixed sequence position and associates these summed contributions with the nucleotide present at that base position in the interpreted input. We refer to these summed contributions as “DeepLIFT interpretation scores”, and all results make comparison to this output of the DeepLIFT interpretation method.

We followed three recommendations of [1] when applying DeepLIFT. First, we calculated contributions of input neurons to the pre-activation (activation before applying final non-linearity) of an output neuron. Second, whenever the output layer used a softmax non-linearity, we mean centered the weights connecting a fixed penultimate layer neuron to the set of output neurons. Third, because we always applied DeepLIFT to sequences classifying one-hot encoded sequence, we used the method of “weight normalization for constrained inputs,” a procedure described in [1], before converting from Keras to DeepLIFT models.

# Implementation of Saliency Map method

We implemented the Saliency Map method of [3], which is a modification of the original method of [4], for the case of one-hot encoded genomic inputs. Briefly, the method captures the extent to which the *i*-th nucleotide in a length L one-hot encoded sequence $\boldsymbol{x}$ (an (Lx4) array with one 1 entry per row and all other entries 0) contributes to the classification encoded by an output neuron by assigning a Saliency Map interpretation score:

(S3.2.1)

$$S_{i}= \sum_{j=1}^{4} \frac{\partial z}{\partial x_{i,j}}{(x}_{i,j}-0)$$

where $z$ denotes the pre-activation of an output neuron. We note that $S_{i}$ can be viewed as (minus) the 1^st^ order Taylor approximation of the change in $z$ when all input neurons are set to 0.

This definition of the Saliency Map interpretation score is capable of reflecting whether a given nucleotide is evidence for or against the classification encoded by the output neuron associated with $z$. We used this definition in interpretation of XOR, motif discovery and nucleosome positioning networks. For calling Saliency Map motif positions we followed [3] by taking the absolute values of the $S_{i}$ defined in (S3.2.1) (see Text S3.4).

# Application of DeepLIFT and Saliency Map to XOR networks

We applied DeepLIFT and Saliency Map interpretation to each of the 30 networks that successfully learned the XOR logic described in Application 1. DeepLIFT contributions and Saliency Map gradients were calculated with respect the pre-activation of the sigmoidal output neuron. For this reason, in plots of DeepLIFT and Saliency Map interpretation scores, positive values should be interpreted as evidence for network classification of the dinucleotide as class 1. Similarly, negative interpretation scores are interpreted as evidence for classification of the dinucleotide as class 0.

We tested DeepLIFT interpretation with two of the sets of references that have been suggested by DeepLIFT’s developers: input neurons set to the frequencies of the nucleotides they encode (denoted “nucleotide frequency reference” in Figure 2) and all input neurons set to 0 (denoted “zeros reference”) in Figure 2.

# Application of DeepLIFT and Saliency Map to motif discovery network

DeepLIFT contributions and Saliency Map gradients were calculated with respect to the pre-activation of the softmax neuron encoding the presence of CTCF binding. To apply DeepLIFT, we set the input neuron reference activations to the frequency of the encoded nucleotide in the test data set (P(A) = P(T) = 0.27 and P(C)=P(G) = 0.23). For each of the 2500 input sequences used to generate Figure 3C and D, we called a DeepLIFT motif at the 19 bp interval with largest mean DeepLIFT interpretation score as defined in Text S3.1. When calling Saliency Map motifs, we followed the method of [3] and called a Saliency Map motif at the 19 bp interval where the mean of the absolute values of the $S_{i}$’s (defined in equation (S3.2.1)) is largest.

# Application of DeepLIFT and Saliency Map to nucleosome positioning network

# Transformation of first layer parameters for one-hot input

DeepLIFT and Saliency Map methods for interpreting ANNs classifying DNA sequence are most clearly articulated for networks trained on one-hot encoded input sequences. Because we trained the nucleosome positioning network of Application 3 on mean centered and scaled one-hot encoded sequence data (Methods), it was necessary to transform the weights and biases of the first convolutional layer of the trained network so that a one-hot encoded sequence input to the ANN with transformed first layer parameters elicited the same network activations at all layers as the corresponding mean centered and scaled sequence input to the untransformed ANN. The transformation was achieved by straightforward rescaling of the weights of convolutional filters and shifting of the associated convolutional biases. We verified that this transformation did not change network activations.

We applied DeepLIFT and Saliency Map interpretation to the transformed network accepting one-hot encoded input. In particular, the transformed network was subjected to all the recommendations for applying DeepLIFT described in Text S3.1. DeepLIFT contributions and Saliency Map gradients were calculated with respect to the pre-activation of the softmax neuron encoding nucleosomal classification.

# Calculation of normalized Fourier amplitudes from DeepLIFT and Saliency Map interpretation scores

# To calculate discrete Fourier transforms of single nucleotide signals from DeepLIFT and Saliency Map interpretation scores, it was necessary to produce for each input sequence a “time series” of 201 values for each type of nucleotide. We achieved this by defining for each interpreted sequence a $\boldsymbol{201}$ x 4 array with entries $\boldsymbol{a}_{\boldsymbol{i,j}}$ where *i* indexes the base position and *j* indexes the nucleotide type (A,C,G,T) and $\boldsymbol{a}_{\boldsymbol{i,j}}$ is given by the DeepLIFT/Saliency Map interpretation score at position *i* if the nucleotide at that position is type *j* or is zero otherwise. For each fixed *j*, we calculated the normalized Fourier amplitudes of the 1-dimensional array $\boldsymbol{a}_{\boldsymbol{i,j}}$. These normalized amplitudes were averaged to produce the plots in Figure 4D.

S3.6 Supplementary References

1. Shrikumar A, Greenside P, Shcherbina A, Kundaje A. Not Just a Black Box: Learning Important Features Through Propagating Activation Differences. 2016;(arXiv:1605.01713 [cs.LG]).

2. Chollet F. Keras: GitHub; 2015. Available from: <https://github.com/fchollet/keras>.

3. Lanchantin J, Singh R, Wang B, Qi Y. Deep Motif Dashboard: Visualizing and Understanding Genomic Sequences Using Deep Neural Networks. Pac Symp Biocomput. 2016;22:254-65. PubMed PMID: 27896980.

4. Simonyan K, Vedaldi A, Zisserman A, editors. Deep Inside Convolutional Networks: Visualising Image Classification Models and Saliency Maps. ICLR Workshop 2014.
