# Supplementary material for: Maximum entropy methods for extracting the learned features of deep neural networks
Source: PLoS Comput Biol. 2017 Oct 30;13(10):e1005836. doi: 10.1371/journal.pcbi.1005836 (PMC5679649; doi:10.1371/journal.pcbi.1005836)
Supplement: S1 Fig — (PDF) [file pcbi.1005836.s004.pdf]

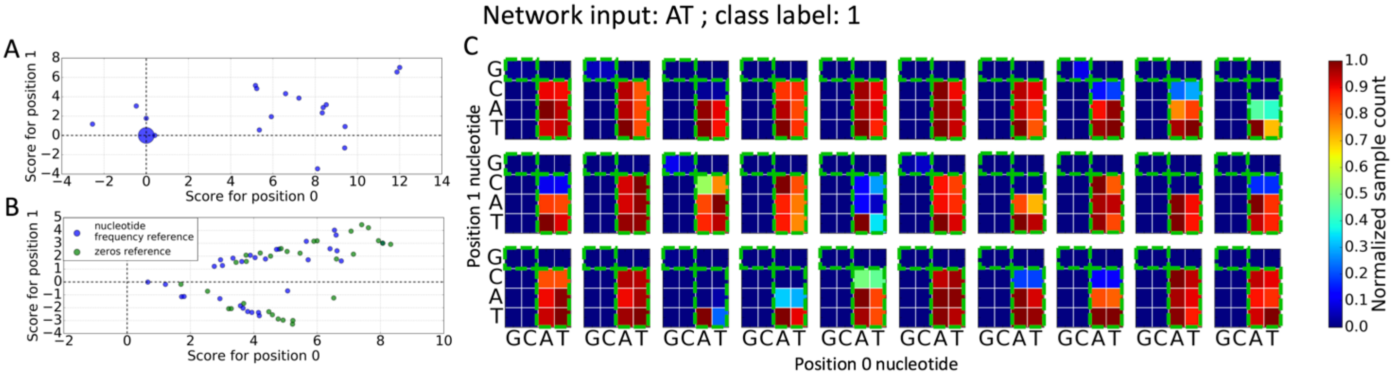

Figure S1.1. Interpretation of AT XOR network input. (A) and (**B**) scatter plot of interpretation scores assigned to the 0th and 1st sequence position by Saliency Map and DeepLIFT interpretation, respectively, for the AT network input. Markers at the origin have size in proportion to number of overlapping data points. Colors in (**B**) indicate DeepLIFT interpretation scores using different reference inputs (see Text S3.3). **(C)** Density of MCMC samples from the distribution (2) for AT input to same networks as in (**A**) and (**B**)**.** Densities are normalized by the most abundant dinucleotide. Green boxes indicate set of dinucleotides assigned to class 1.

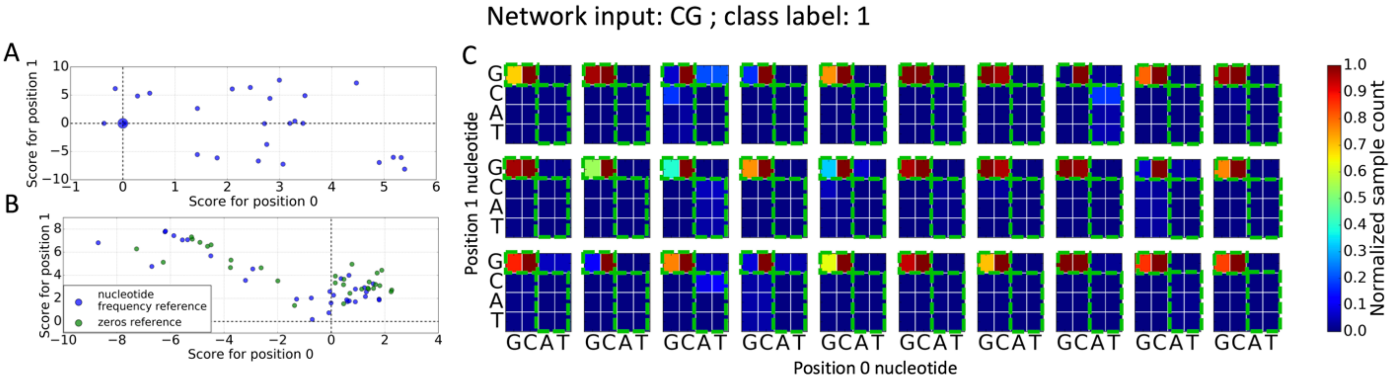

Figure S1.2. Interpretation of CG XOR network input. (A) and (**B**) scatter plot of interpretation scores assigned to the 0th and 1st sequence position by Saliency Map and DeepLIFT interpretation, respectively, for the CG network input. Markers at the origin have size in proportion to number of overlapping data points. Colors in (**B**) indicate DeepLIFT interpretation scores using different reference inputs (see Text S3.3). **(C)** Density of MCMC samples from the distribution (2) for CG input to same networks as in (**A**) and (**B**)**.** Densities are normalized by the most abundant dinucleotide. Green boxes indicate set of dinucleotides assigned to class 1.

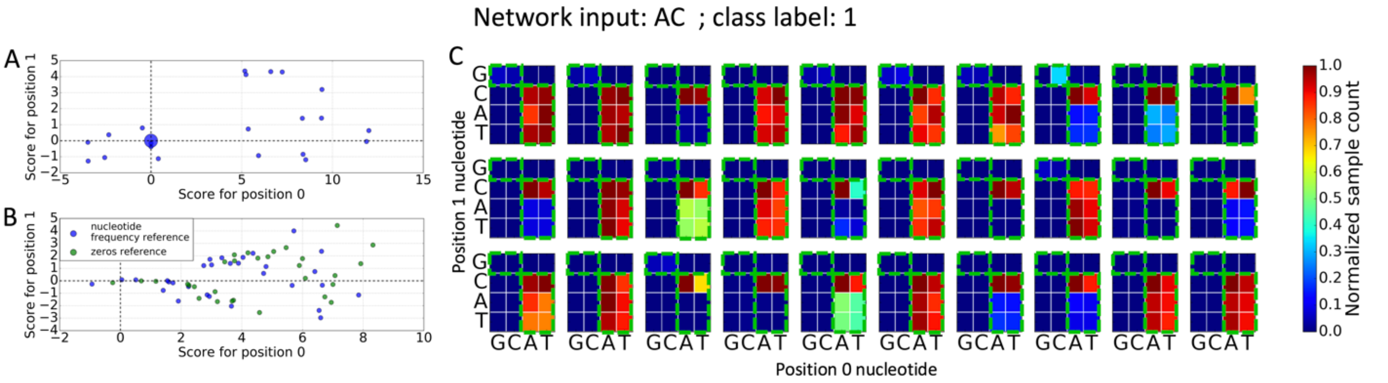

Figure S1.3. Interpretation of AC XOR network input. (**A**) and (**B**) scatter plot of interpretation scores assigned to the 0th and 1st sequence position by Saliency Map and DeepLIFT interpretation, respectively, for the AC network input. Markers at the origin have size in proportion to number of overlapping data points. Colors in (**B**) indicate DeepLIFT interpretation scores using different reference inputs (see Text S3.3). **(C)** Density of MCMC samples from the distribution (2) for AC input to same networks as in (**A**) and (**B**)**.** Densities are normalized by the most abundant dinucleotide. Green boxes indicate set of dinucleotides assigned to class 1.

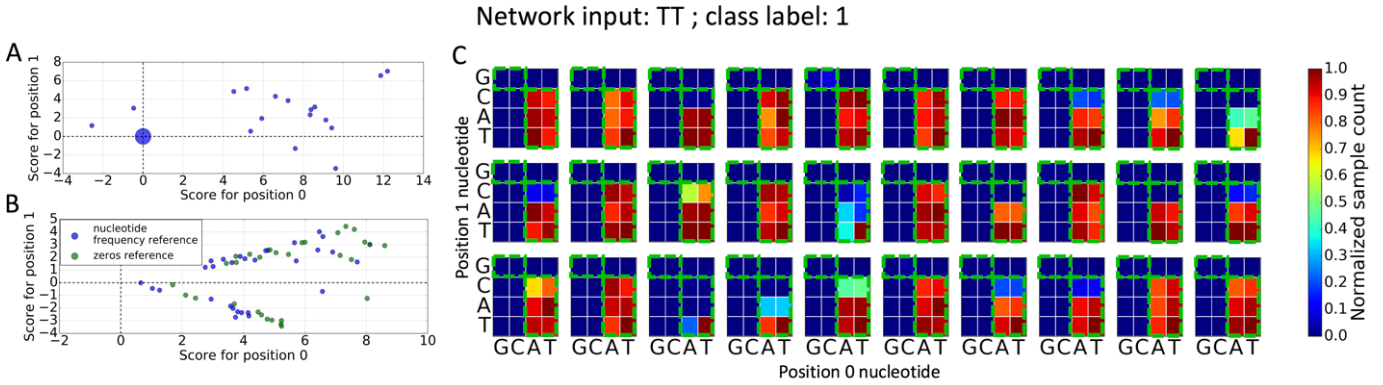

Figure S1.4. Interpretation of TT XOR network input. (**A**) and (**B**) scatter plot of interpretation scores assigned to the 0th and 1st sequence position by Saliency Map and DeepLIFT interpretation, respectively, for the TT network input. Markers at the origin have size in proportion to number of overlapping data points. Colors in (**B**) indicate DeepLIFT interpretation scores using different reference inputs (see Text S3.3). **(C)** Density of MCMC samples from the distribution (2) for TT input to same networks as in (**A**) and (**B**)**.** Densities are normalized by the most abundant dinucleotide. Green boxes indicate set of dinucleotides assigned to class 1.

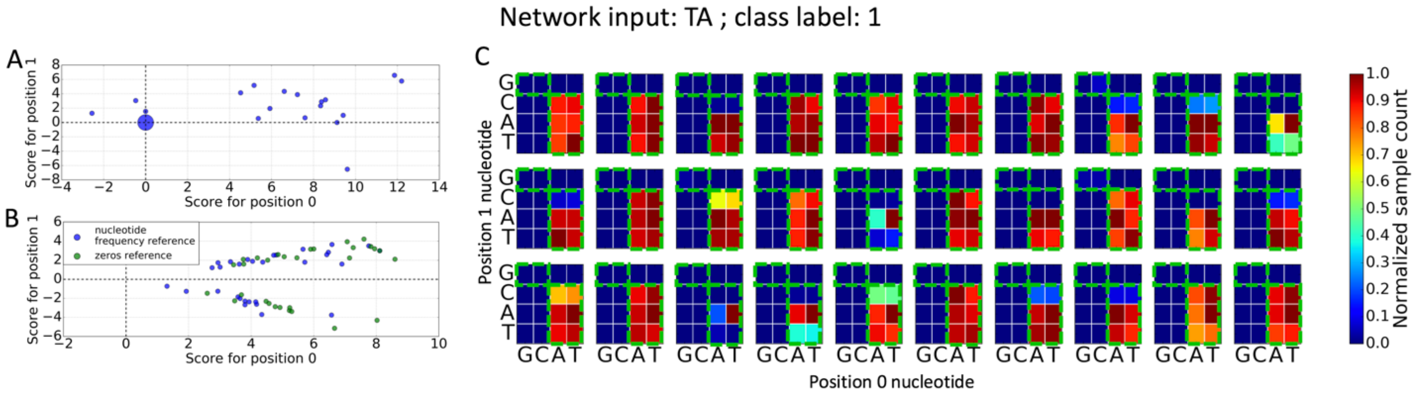

Figure S1.5. Interpretation of TA XOR network input. (**A**) and (**B**) scatter plot of interpretation scores assigned to the 0th and 1st sequence position by Saliency Map and DeepLIFT interpretation, respectively, for the TA network input. Markers at the origin have size in proportion to number of overlapping data points. Colors in (**B**) indicate DeepLIFT interpretation scores using different reference inputs (see Text S3.3). **(C)** Density of MCMC samples from the distribution (2) for TA input to same networks as in (**A**) and (**B**)**.** Densities are normalized by the most abundant dinucleotide. Green boxes indicate set of dinucleotides assigned to class 1.

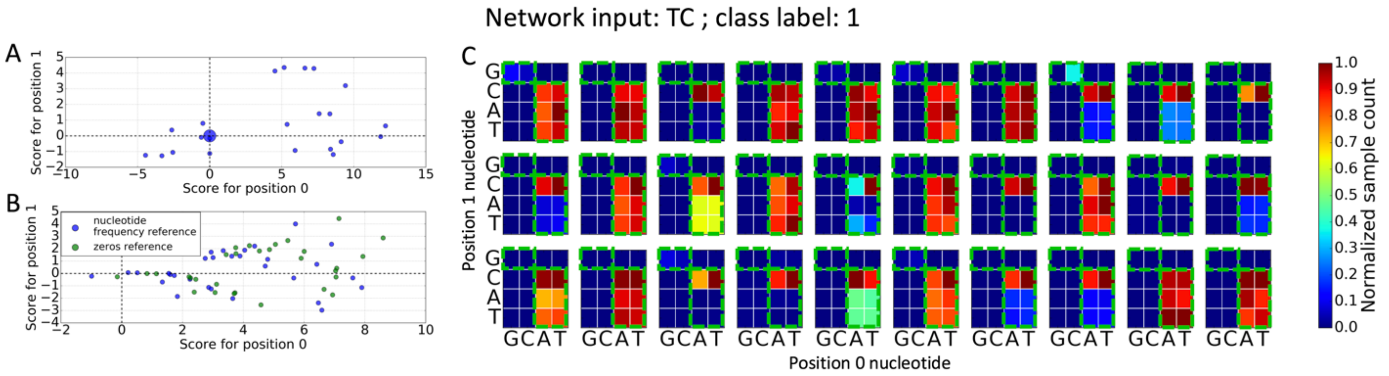

Figure S1.6. Interpretation of TC XOR network input. (**A**) and (**B**) scatter plot of interpretation scores assigned to the 0th and 1st sequence position by Saliency Map and DeepLIFT interpretation, respectively, for the TC network input. Markers at the origin have size in proportion to number of overlapping data points. Colors in (**B**) indicate DeepLIFT interpretation scores using different reference inputs (see Text S3.3). **(C)** Density of MCMC samples from the distribution (2) for TC input to same networks as in (**A**) and (**B**)**.** Densities are normalized by the most abundant dinucleotide. Green boxes indicate set of dinucleotides assigned to class 1.

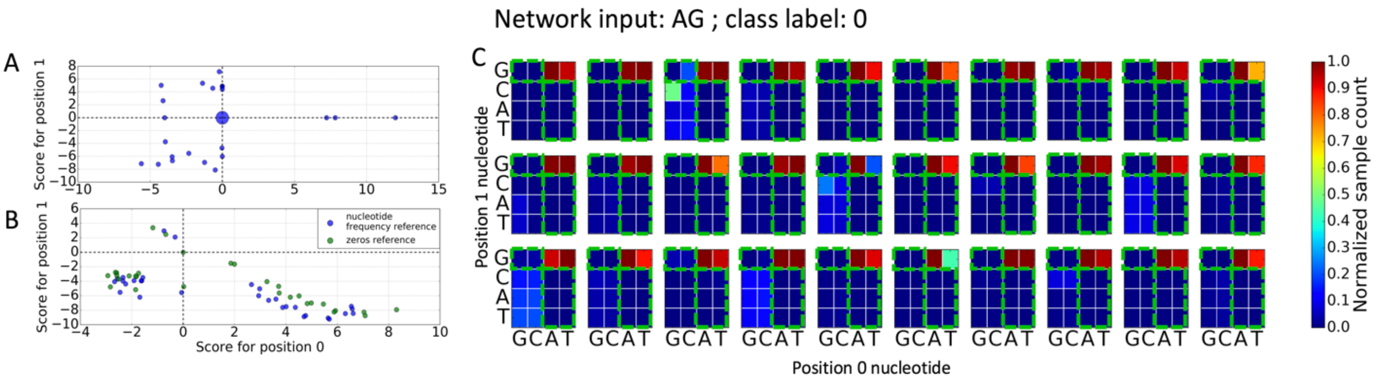

Figure S1.7. Interpretation of AG XOR network input. (A) and (**B**) scatter plot of interpretation scores assigned to the 0th and 1st sequence position by Saliency Map and DeepLIFT interpretation, respectively, for the AG network input. Markers at the origin have size in proportion to number of overlapping data points. Colors in (**B**) indicate DeepLIFT interpretation scores using different reference inputs (see Text S3.3). Note that, because this dinucleotide is correctly assigned to class 0 by the network, negative Salience Map and DeepLIFT scores indicate evidence for the network's classification. **(C)** Density of MCMC samples from the distribution (2) for AG input to same networks as in (**A**) and (**B**)**.** Densities are normalized by the most abundant dinucleotide. Green boxes indicate set of dinucleotides assigned to class 1.

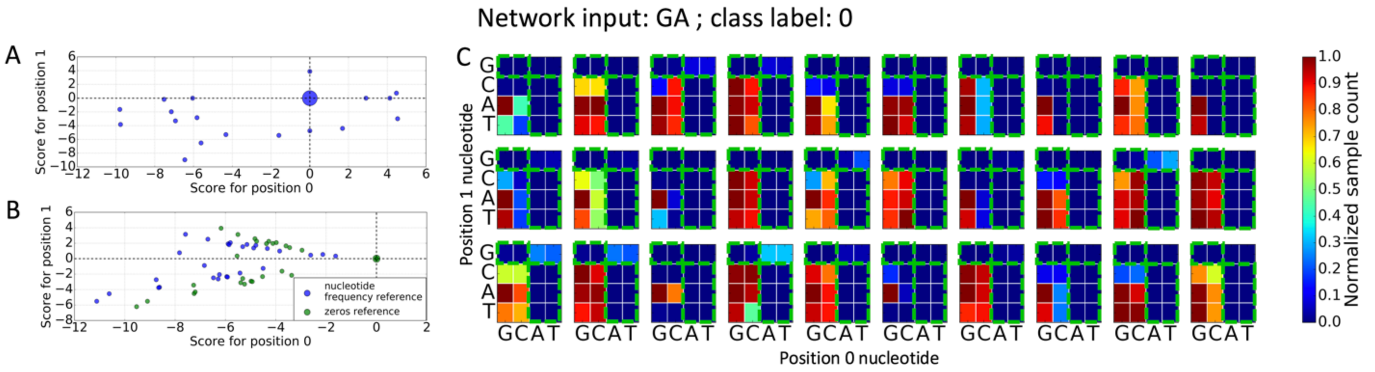

Figure S1.8. Interpretation of GA XOR network input. (A) and (**B**) scatter plot of interpretation scores assigned to the 0th and 1st sequence position by Saliency Map and DeepLIFT interpretation, respectively, for the GA network input. Markers at the origin have size in proportion to number of overlapping data points. Colors in (**B**) indicate DeepLIFT interpretation scores using different reference inputs (see Text S3.3). Note that, because this dinucleotide is correctly assigned to class 0 by the network, negative Salience Map and DeepLIFT scores indicate evidence for the network's classification. **(C)** Density of MCMC samples from the distribution (2) for GA input to same networks as in (**A**) and (**B**)**.** Densities are normalized by the most abundant dinucleotide. Green boxes indicate set of dinucleotides assigned to class 1.

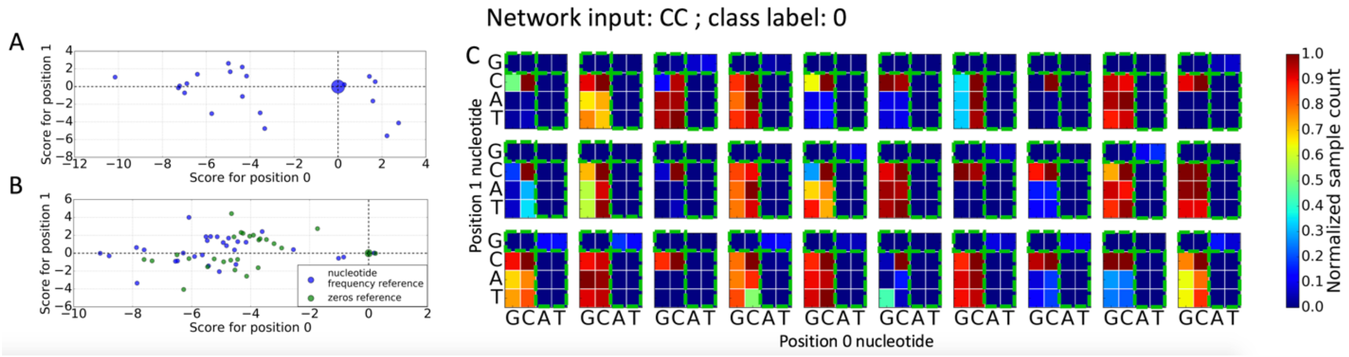

Figure S1.9. Interpretation of CC XOR network input. (A) and (**B**) scatter plot of interpretation scores assigned to the 0th and 1st sequence position by Saliency Map and DeepLIFT interpretation, respectively, for the CC network input. Markers at the origin have size in proportion to number of overlapping data points. Colors in (**B**) indicate DeepLIFT interpretation scores using different reference inputs (see Text S3.3). Note that, because this dinucleotide is correctly assigned to class 0 by the network, negative Salience Map and DeepLIFT scores indicate evidence for the network's classification. **(C)** Density of MCMC samples from the distribution (2) for CC input to same networks as in (**A**) and (**B**)**.** Densities are normalized by the most abundant dinucleotide. Green boxes indicate set of dinucleotides assigned to class 1.

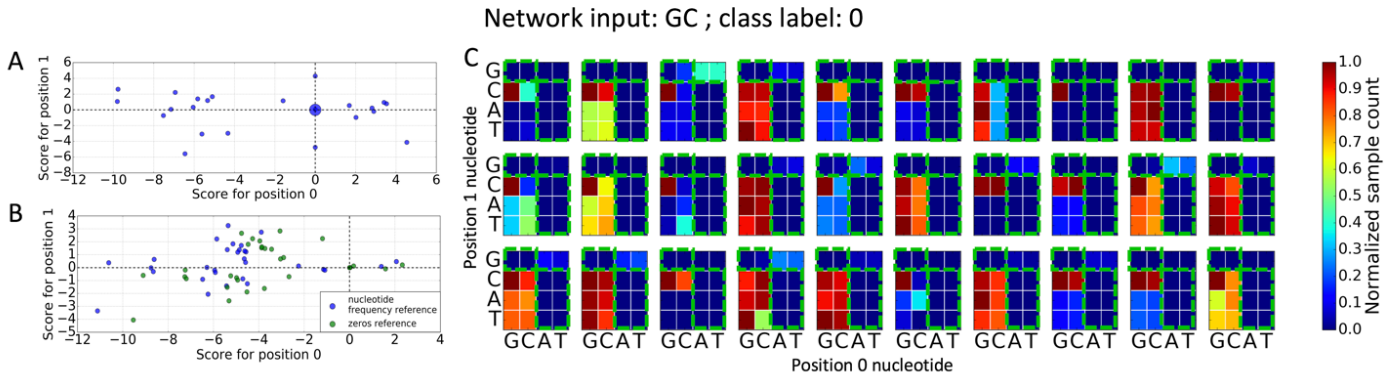

Figure S1.10, Interpretation of GC XOR network input. (A) and (**B**) scatter plot of interpretation scores assigned to the 0th and 1st sequence position by Saliency Map and DeepLIFT interpretation, respectively, for the GC network input. Markers at the origin have size in proportion to number of overlapping data points. Colors in (**B**) indicate DeepLIFT interpretation scores using different reference inputs (see Text S3.3). Note that, because this dinucleotide is correctly assigned to class 0 by the network, negative Salience Map and DeepLIFT scores indicate evidence for the network's classification. **(C)** Density of MCMC samples from the distribution (2) for GC input to same networks as in (**A**) and (**B**)**.** Densities are normalized by the most abundant dinucleotide. Green boxes indicate set of dinucleotides assigned to class 1.

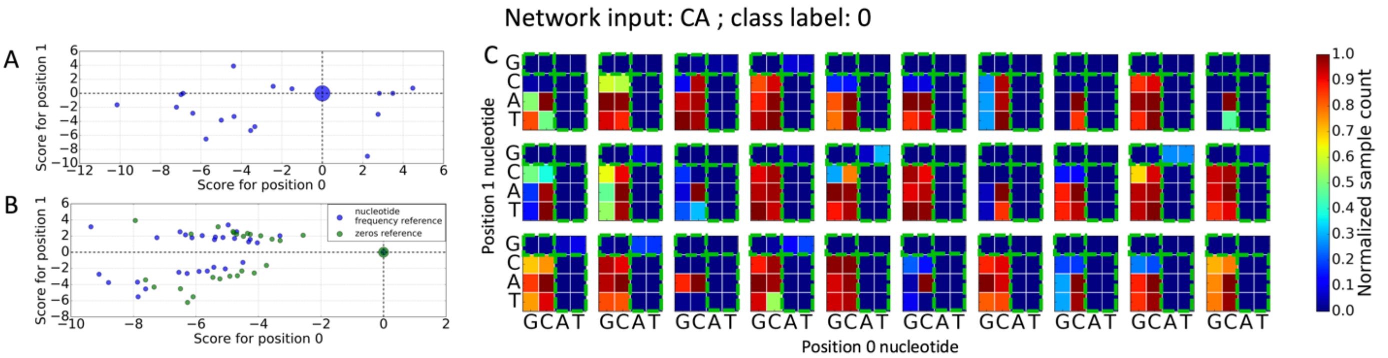

Figure S1.11. Interpretation of CA XOR network input. (A) and (**B**) scatter plot of interpretation scores assigned to the 0th and 1st sequence position by Saliency Map and DeepLIFT interpretation, respectively, for the CA network input. Markers at the origin have size in proportion to number of overlapping data points. Colors in (**B**) indicate DeepLIFT interpretation scores using different reference inputs (see Text S3.3). Note that, because this dinucleotide is correctly assigned to class 0 by the network, negative Salience Map and DeepLIFT scores indicate evidence for the network's classification. **(C)** Density of MCMC samples from the distribution (2) for CA input to same networks as in (**A**) and (**B**)**.** Densities are normalized by the most abundant dinucleotide. Green boxes indicate set of dinucleotides assigned to class 1.

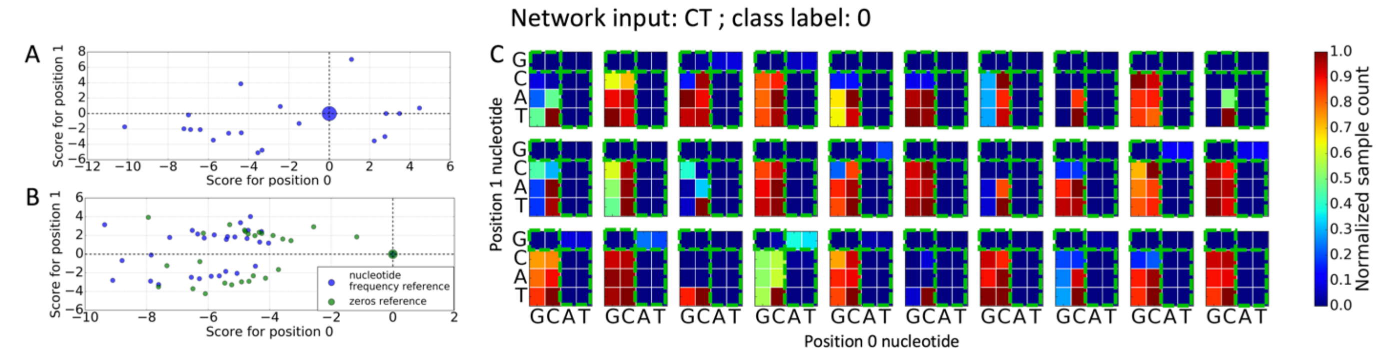

Figure S1.12. Interpretation of CT XOR network input. (**A**) and (**B**) scatter plot of interpretation scores assigned to the 0th and 1st sequence position by Saliency Map and DeepLIFT interpretation, respectively, for the CT network input. Markers at the origin have size in proportion to number of overlapping data points. Colors in (**B**) indicate DeepLIFT interpretation scores using different reference inputs (see Text S3.3). Note that, because this dinucleotide is correctly assigned to class 0 by the network, negative Salience Map and DeepLIFT scores indicate evidence for the network's classification. **(C)** Density of MCMC samples from the distribution (2) for CT input to same networks as in (**A**) and (**B**)**.** Densities are normalized by the most abundant dinucleotide. Green boxes indicate set of dinucleotides assigned to class 1

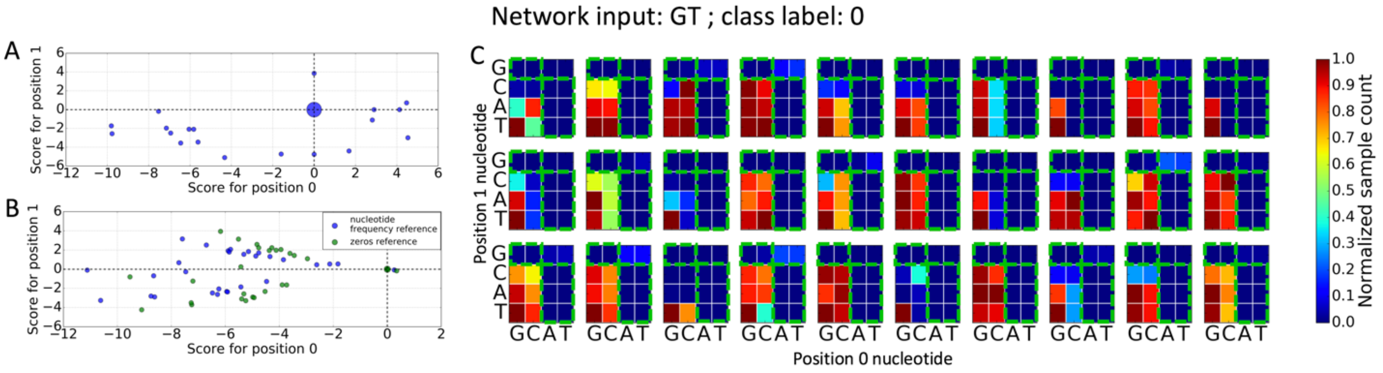

Figure S1.13. Interpretation of GT XOR network input. (**A**) and (**B**) scatter plot of interpretation scores assigned to the 0th and 1st sequence position by Saliency Map and DeepLIFT interpretation, respectively, for the GT network input. Markers at the origin have size in proportion to number of overlapping data points. Colors in (**B**) indicate DeepLIFT interpretation scores using different reference inputs (see Text S3.3). Note that, because this dinucleotide is correctly assigned to class 0 by the network, negative Salience Map and DeepLIFT scores indicate evidence for the network's classification. **(C)** Density of MCMC samples from the distribution (2) for GT input to same networks as in (**A**) and (**B**)**.** Densities are normalized by the most abundant dinucleotide. Green boxes indicate set of dinucleotides assigned to class 1.

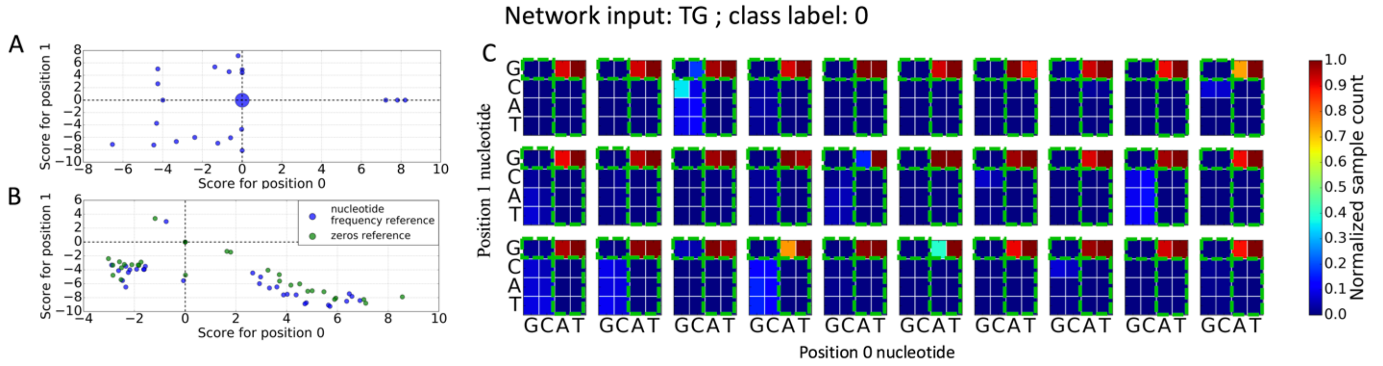

Figure S1.14. Interpretation of TG XOR network input. (**A**) and (**B**) scatter plot of interpretation scores assigned to the 0th and 1st sequence position by Saliency Map and DeepLIFT interpretation, respectively, for the TG network input. Markers at the origin have size in proportion to number of overlapping data points. Colors in (**B**) indicate DeepLIFT interpretation scores using different reference inputs (see Text S3.3). Note that, because this dinucleotide is correctly assigned to class 0 by the network, negative Salience Map and DeepLIFT scores indicate evidence for the network's classification. **(C)** Density of MCMC samples from the distribution (2) for TG input to same networks as in (**A**) and (**B**)**.** Densities are normalized by the most abundant dinucleotide. Green boxes indicate set of dinucleotides assigned to class 1.
