# Supplementary material for: Maximum entropy methods for extracting the learned features of deep neural networks
Source: PLoS Comput Biol. 2017 Oct 30;13(10):e1005836. doi: 10.1371/journal.pcbi.1005836 (PMC5679649; doi:10.1371/journal.pcbi.1005836)
Supplement: S2 Fig — (PDF) [file pcbi.1005836.s005.pdf]

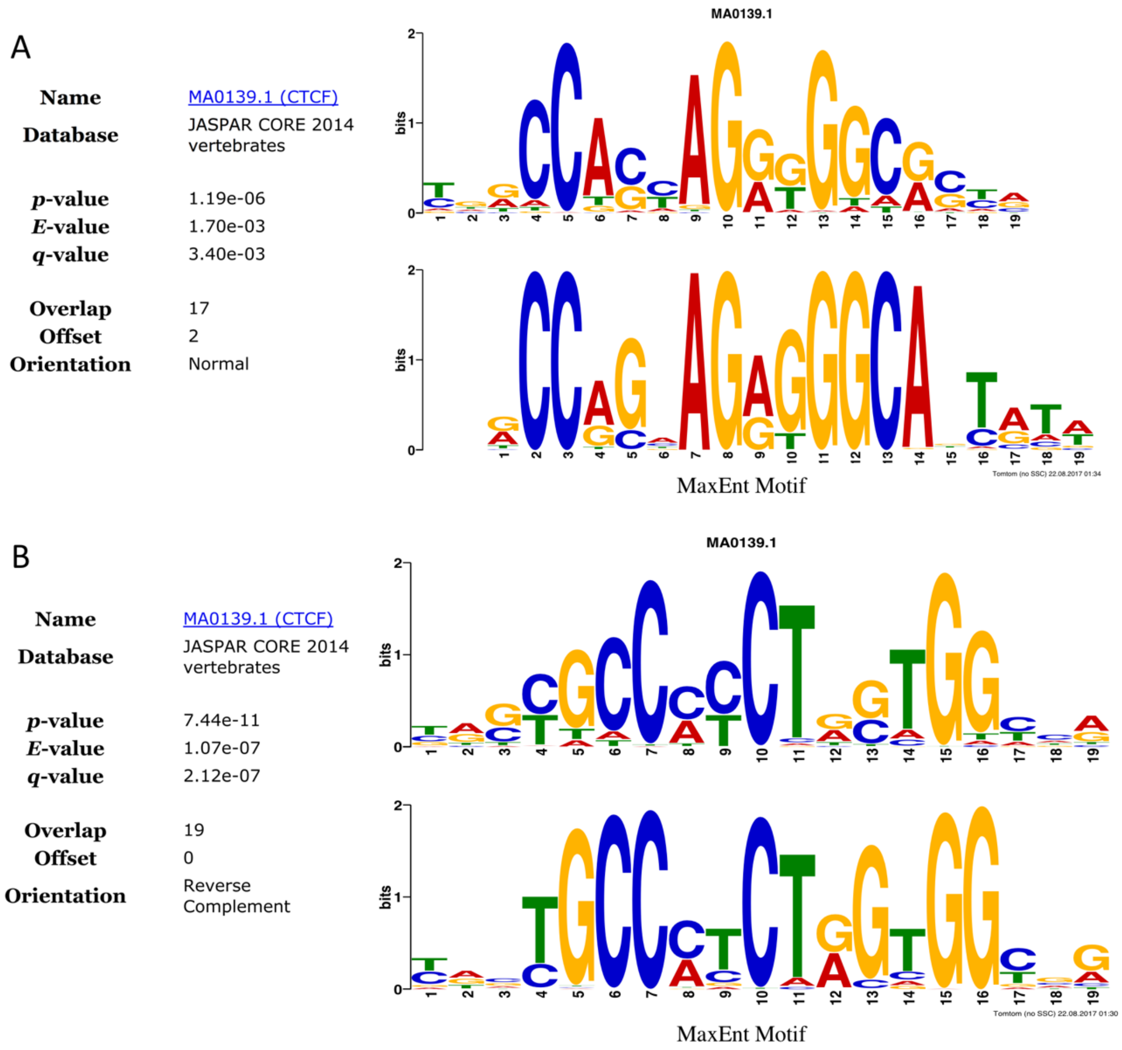

Figure S2. Top hits for Tomtom database query with MaxEnt motifs. (A) MaxEnt motif (bottom) and best database match (top) for the MaxEnt motif in Figure 3A. Tomtom Motif agreement statistics are shown on the left. **(B)** Same as (A) but for the MaxEnt motif in Figure 3B
