# Supplementary material for: Maximum entropy methods for extracting the learned features of deep neural networks
Source: PLoS Comput Biol. 2017 Oct 30;13(10):e1005836. doi: 10.1371/journal.pcbi.1005836 (PMC5679649; doi:10.1371/journal.pcbi.1005836)
Supplement: S3 Fig — (PDF) [file pcbi.1005836.s006.pdf]

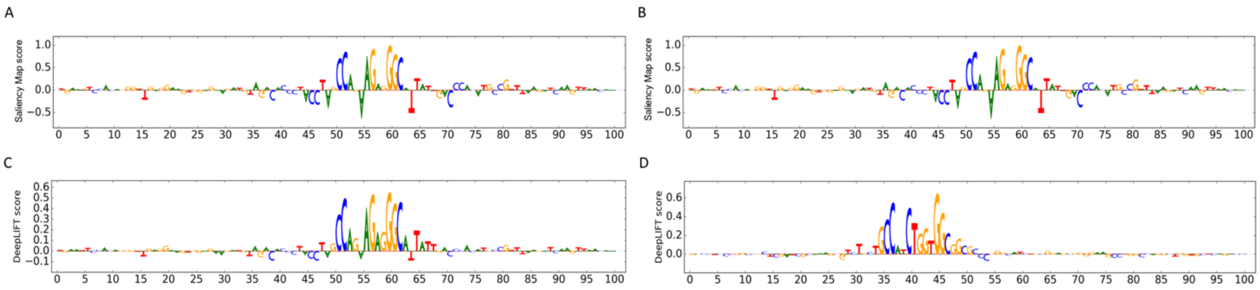

Figure S3. Interpretation of CTCF-bound sequences. (**A**) and (**B**) Saliency map interpretation score for the sequence input analyzed by MaxEnt interpretation in Fig. 3 (A) and (B), respectively. (Representation of Saliency Map score uses code from [1]). (**C**) and (**D**) DeepLIFT interpretation score for the sequence input analyzed by MaxEnt interpretation in Fig. 3 (A) and (B), respectively. (Representation of DeepLIFT score uses code from [1])

Supplementary Reference for Figure S3

1. Shrikumar A. DeepLIFT 2017. GitHub. Available from: https://github.com/kundajelab/deeplift.
