# Supplementary material for: Maximum entropy methods for extracting the learned features of deep neural networks
Source: PLoS Comput Biol. 2017 Oct 30;13(10):e1005836. doi: 10.1371/journal.pcbi.1005836 (PMC5679649; doi:10.1371/journal.pcbi.1005836)
Supplement: S4 Fig — (PDF) [file pcbi.1005836.s007.pdf]

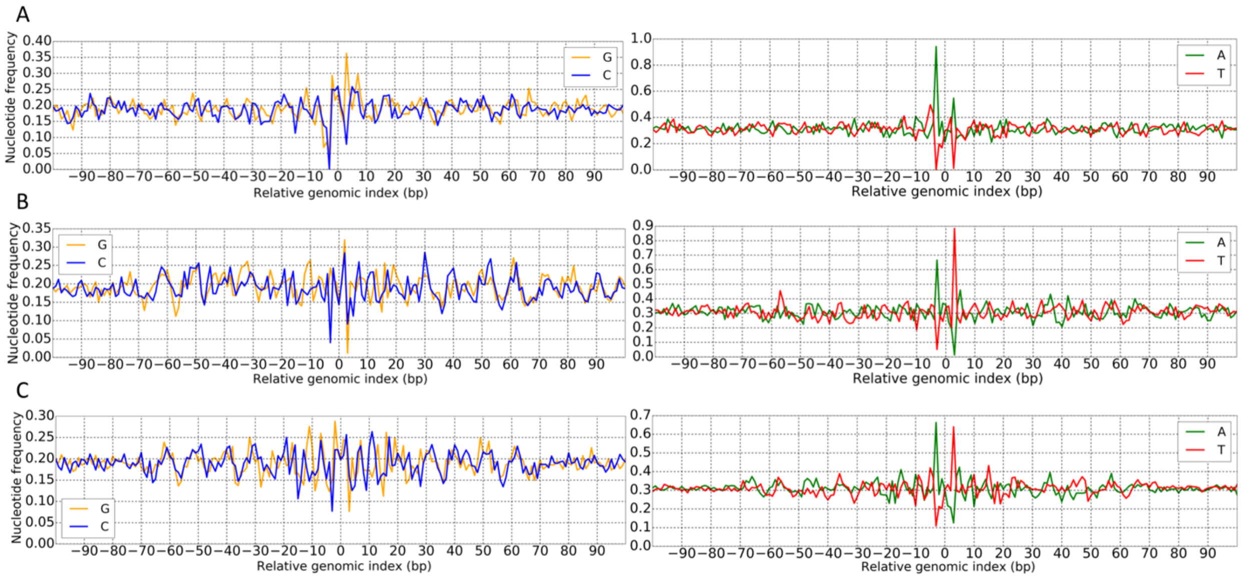

Figure S4. Additional plots of nucleosome single nucleotide frequencies. (A,B) Single nucleotide frequencies of samples from MaxEnt distributions for two additional nucleosomal sequences correctly classified by the ANN. These sequences exemplify reduced or in incomplete 10 bp periodic nucleotide content in MaxEnt samples, relative to Figure 4A. (**C**) Single nucleotide frequencies averaged over 2500 interpreted nucleosomal sequences.
