# Supplementary material for: Maximum entropy methods for extracting the learned features of deep neural networks
Source: PLoS Comput Biol. 2017 Oct 30;13(10):e1005836. doi: 10.1371/journal.pcbi.1005836 (PMC5679649; doi:10.1371/journal.pcbi.1005836)
Supplement: S5 Fig — (PDF) [file pcbi.1005836.s008.pdf]

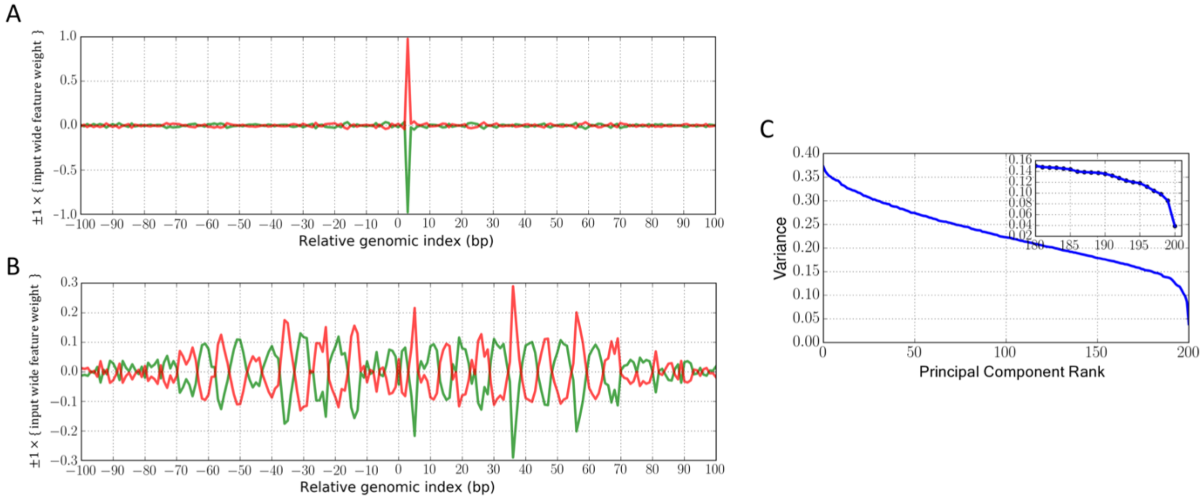

Figure S5. Examples of learned low-variance nucleosome features. Plots of the weights, c_i, of the input-wide features with largest **(A)** and second largest **(B)** feature importance scores δ under the approximation (8). Red and green traces are reflections over the $c_i = 0$ axis, shown because δ is unaffected by collective change in sign of the c_i's. **(C)** The decay in variance of PC vectors in PCA decomposition used to determine input-wide features plotted in (A) and (B). The sharp decay in variance signals a sharp rise in importance score δ.
